# AMMO-Prot: amine system project 3D-model finder

**DOI:** 10.1186/1471-2105-9-S4-S5

**Published:** 2008-04-25

**Authors:** Ismael Navas-Delgado, Raúl Montañez, Almudena Pino-Ángeles, Aurelio A Moya-García, José Luis Urdiales, Francisca Sánchez-Jiménez, José F Aldana-Montes

**Affiliations:** 1 Computer Languages and Computing Science Department, University of Málaga, Málaga, 29071, Spain; 2 Molecular Biology and Biochemistry Department. University of Málaga, and CIBER of Rare Diseases, National Institute of Health, Málaga, 29071, Spain

## Abstract

**Background:**

Amines are biogenic amino acid derivatives, which play pleiotropic and very important yet complex roles in animal physiology. For many other relevant biomolecules, biochemical and molecular data are being accumulated, which need to be integrated in order to be effective in the advance of biological knowledge in the field. For this purpose, a multidisciplinary group has started an ontology-based system named the Amine System Project (ASP) for which amine-related information is the validation bench.

**Results:**

In this paper, we describe the Ontology-Based Mediator developed in the Amine System Project () using the infrastructure of Semantic Directories, and how this system has been used to solve a case related to amine metabolism-related protein structures.

**Conclusions:**

This infrastructure is used to publish and manage not only ontologies and their relationships, but also metadata relating to the resources committed with the ontologies. The system developed is available at .

## Background

Over the last few years the internet has become a large information repository that is accessed manually in the vast majority of cases. However, the flexibility and openness of the internet in computer systems is lacking when software applications are connected. The main aim of the Semantic Web is to automatically perform tasks done in the current Contents Web. This will be done by making explicit the semantics of the contents, thereby providing unambiguous knowledge to Web documents and applications. For any field of knowledge, and particularly in Life Sciences, research on Semantic Web infrastructures and applications can be especially helpful to improve efficiency in the finding, collection and organization of data stored in the growing number of resources which make their semantics explicit.

In the context of Life Sciences, the frame of Systems Biology is being merged [1]. It is supported by all high-throughput methods which generate large amounts of data that cannot be covered simply by the human mind. This field includes a wide variety of concepts and methods but, in general, it can be considered the analysis of living systems, through the study of the relationships among the elements in response to genetic or environmental perturbations, with the ultimate goal of understanding the system as a whole. A “system” can be considered at different levels, from a metabolic pathway or gene regulatory network to a cell, tissue, organism or ecosystem. The number of information repositories and services for biological elements (molecules, cells, etc) is growing exponentially. Consequently, Systems Biology is the prototype of a knowledge-intensive application domain for which the Semantic Web should be particularly interesting.

Initially, our system will comprise the biochemistry, molecular biology and physiopathology related to amines. We are using this system to develop, validate and apply our infrastructure and are focusing at this stage mainly on amines derived from cationic amino acids. They are histamine and polyamines, which have been the main area of research in our laboratory for the last 15 years [2-6]. Their biosynthetic pathways start with the alfa-decarboxylation of their respective amino acid precursors by enzymes that cannot be easily purified from their native sources.

These metabolic pathways also involve different proteins with transferase, oxidase and dehydrogenase activities that are not fully-characterized yet [7]. However, these pathways are considered well-defined modules of secondary nitrogen metabolism, with minimum input and output from/to other biochemical modules, where most of their components have at least been defined. Concerning their physiological roles, these compounds play pleiotropic roles in human and animal physiology, being involved in many different physiopathological conditions. Histamine has been related to allergies and inflammation, gastric acid secretion, neurotransmission and tumour progression [8]. Polyamines are essential for cell growth and their levels are closely linked to the survival of every living cell. Thus, their metabolism is a promising target for anti-proliferative strategies of chemoprevention and the treatment of cancer and parasitic infections. In addition, they also act as differentiation and neurotransmission regulators [9]. From this, we can deduce that the choice of these biomodules as pilots for our integration project has the following advantages:

• The physiopathological problems (cancer, allergic and inflammatory processes, as well as many other emerging and rare diseases) associated with these compounds and their metabolic pathways are global and affect most of humanity at some stage of their lives. These circumstances explain the growing interest in the information obtained from this project.

• The information on amine-related processes is dispersed among specific bibliographies of many different scientific areas: Oncology, Immunology and Haematology, Neurobiology, Pharmacology and Basic Biophysics, Biochemical and Molecular Biology Research, which makes manual integration of all the valuable data more difficult. Thus, this project can contribute to the efficiency of the scientific advances in the field.

• Many molecular questions still remain to be solved with respect to the structure/function relationships of their components, the extracellular and intracellular communication pathways involved in regulating these metabolic pathways and the physiological effects of these amines. Therefore, the integration of information (as a part of Systems Biology techniques) can provide the best perspective for analyzing these complex molecular relationships and networks established in living systems [1].

• Many of the ontologies and tools developed throughout this pilot project could be easily extended to solve other biological problems, as deduced from the most recent bibliography [10-13].

• Finally, this interdisciplinary group combines experimental and bioinformatics approaches for studies in this field. Thus, any result or prediction can be easily checked and even experimentally validated [2-4].

We propose a generic infrastructure for publishing and managing knowledge and information on the Semantic Web. This infrastructure is based on a resource directory, called Semantic Directory, containing information about web resource semantics. This paper focuses on the resolution of data integration problems by concentrating on our proposal of a generic infrastructure architecture. We have developed an Ontology-Based Mediator, which has been applied to solve a data integration problem in this biological domain (Amine System Project, ). The main advantage of this mediator is that data sources and services can be easily plugged into the system (by describing the semantics with respect to registered ontologies). Furthermore, the semantic description is a simple process, thanks to the use of the proposed infrastructure (see Section Ontology-Based Mediator: An application example). This mediator is currently available through a use case published as a Web site [14].

### Related works

This section describes some related works which are grouped depending on their goals. Thus, we first describe data integration systems (from traditional to ontology-based ones, ending with those focused on bioinformatics resources). The second group is composed of systems that have been developed to solve biological problems, and which provide solutions from different points of view: Web Services, Workflows, and Web Portals.

Our system is a data integration proposal with a test Web interface, which uses ontologies to describe resource semantics and these resources are published as Web Services. The workflow described in the use case has been hand coded (but each step of the workflow is a query that is automatically solved), and in the future, it will be interesting to include workflow management capabilities.

Our main goal is to provide an infrastructure for interoperating applications in the Semantic Web. This infrastructure can be used to build different kinds of applications, so we have developed as a use case the Ontology-Based Mediation system, a system for locating Semantic Web Services [15] and an ontology clustering algorithm [16]. In this paper, we focus on data integration solutions. Thus, we have proposed a mediation architecture based on the wrapper-mediator approach, which has some interesting characteristics:

• It follows an ontology-based approach, in which the AMMO ontology is used as integration schema, but we have also used this ontology to perform basic reasoning processes (class-subclass inference).

• Wrappers are published as Web Services to enable their distribution and use in different applications.

• The mediator is divided into components to enable its extension by including new components (such as query optimization algorithms).

• Information about relationships between resources and the AMMO ontology is distributed using a generic infrastructure for Semantic Web applications.

These characteristics have been proposed in previous works, and have been successfully combined to build a useful application in Systems Biology for solving real scientific problems. The use of this application by ASP members has provided them with an easy-to-use way of solving daily tasks by integrating different data sources. Furthermore, the system provides an intuitive interface, in which the user can click on enzymes in a specific metabolic pathway. This approach can be extended to other metabolic pathways, and can include specialized data sources as KEGG, Reactome, etc. (in which we are currently working).

#### Data integration approaches

Data integration systems are formally defined as a triple <G,S,M> where G is the global (or mediated) schema, S is the heterogeneous set of source schemas, and M is the mapping that maps queries between the source and the global schemas. Both G and S are expressed in languages over alphabets comprised of symbols for each of their respective relationships. The mapping M consists of assertions between queries over G and queries over S. When users send queries to the data integration system, they describe those queries over G and the mapping then asserts connections between the elements in the global schema and the source schemas.

The most important proposal to solve the data integration problem is the wrapper/mediator architecture. In this architecture, a mediator (an intermediate virtual database with a schema G according to a previous definition of the data integration system) is established between data sources (with a set of schemas S) and applications. A wrapper is a data source interface that translates data into a common data model used by the mediator. The user accesses the data sources through one or several mediator systems which present high-level abstractions (views) of combinations of source data. The user does not know where the data comes from but is able to retrieve it using a common mediator query language.

Mediator-based integration has query translation as its main task. A mediator in our context is an application that has to solve queries formulated by the user at runtime in terms of either a single or an integrated schema. These queries are re-written in terms of the data source schemas in order to delegate the query resolution to the data sources. Thus, expressing the relationships between the integrated schema and the data source schemas is a crucial step in the mediation system development. The two main approaches for determining the relationships (mapping) between the integration schema (G) and the data source schemas (S) are: global-as-view (GAV) and local-as-view (LAV)[17].

In GAV, each element in the integration schema should be described in terms of a view (a query) over the data sources. In other words, the mapping makes it explicit how to retrieve data from several elements in the integration schema. This approach is effective when the set of data sources is stable (i.e., it remains relatively unchanged).

It is noteworthy that elements in the integration schema are defined in terms of the data sources, so the addition of a new data source implies the redefinition of some elements in the integration schema. This approach benefits from easier rewriting methods.

In LAV, each element in the data source schemas should be described in terms of the integration schema. This kind of approach is effective when the integration schema is stable and well established in the domain/application. In this case, the extension of the system is easy because it only implies adding the description of the new data source in terms of the integration schema. This approach implies a more difficult query reformulation and evaluation, which contrasts with the benefits of greater scalability.

#### Data integration systems

Data integration systems [18-22] deal with problems that could be solved with the infrastructure presented in this paper. Thus, we propose to relate the semantics (domain ontologies), with the resources' data schemas using mappings, as is done in mediation applications. Our proposal uses this information to solve a wide range of problems, in which mediation is a sub-range. Consequently, it is feasible to develop new information integration applications, by adding new components able to solve specific tasks.

The wrapper-mediator approach provides an interface to a group of (semi) structured data sources, combining their local schemas into a global one and integrating the information of local sources. Therefore, the views of the data that mediators offer are coherent. These mediators perform semantic reconciliation of the common data model representations provided by the wrappers. Some good examples of wrapper-mediator systems are TSIMMIS [23] and Manifold [24]. Several improvements have been made on traditional mediators. One of the most important is the use of standard representation languages, like XML. Thus, the MIX [25] (the successor to the TSIMMIS project) and MOCHA [26] projects are XML-based. However, these kinds of systems are usually built as monolithic systems in which reuse is not possible. Besides, the metadata used to integrate the data sources is not made explicit, so the relationships between resources and integration schemas are not public. This implies that the provenance of the integrated data is unavailable to the users. Our proposal allows this knowledge to be available. The publication of the different components as Web Services therefore makes it possible to reuse them.

The next level of abstraction on Web integration corresponds to ontology-based systems. Their main advantage over mediators is their capacity to manage schemas that are unknown a priori. This is achieved by means of a mechanism that allows contents and query capabilities of the data source to be described declaratively. OBSERVER [27] uses different ontologies to represent data source information. Users explicitly select the ontology to be used for query evaluation. The existence of mappings between ontologies allows the user to change the ontology initially selected. The main disadvantage is that the wrappers are developed for a specific mediator, so they cannot be reused in other mediators. Model-Based Mediation [28] is a paradigm for data integration in which data sources can be integrated, using auxiliary expert knowledge. This knowledge includes information about the domain and is the glue that joins data source schemas together. The expert knowledge is captured in a data structure called Knowledge Map. In Model-Based Mediation, the mediation architecture is extended, taking data sources from the data level without semantics to the conceptual model level. This architecture introduces semantics into data sources and mediators, but it is not published nor is it accessible to agents or applications. Mediators are monolithic systems and they are strongly coupled to wrappers, limiting dynamic integration and interoperability.

In the specific field of biological data there are the following examples: TAMBIS [29], BioDataServer [30], KIND [31], BioZoom [32], BioKleisli [33], DiscoveryLink [34], BioBroker [35] and BioMoby [36].

#### Web Services based systems

BioMoby [36] is a project with the goal of producing an open-source, simple, extensible platform to enable the discovery, representation, integration, and retrieval of biological data from widely disparate data hosts and analysis services. In this platform, data and data analysis tools (for analyzing or transforming data) are distributed in Web Services. Resources are registered in a central server called MOBY central. BioMOBY objects are lightweight XML coded data used as query input and output values.

In summary, the primary components of this infrastructure are MOBY Services (bioinformatics software tools), MOBY Objects (input and output data for the services) and MOBY Central (a register of all resources). This system also offers Object and Service hierarchies in order to classify information and services, helping users to understand the meaning of data required by services. This proposal also includes how to easily develop web services in order to access biological data. Although it introduces the use of web services, it is not exactly an integration architecture, because it is not possible to solve problems directly, but requires access to different databases or services. Furthermore, this proposal does not provide a workflow definition and execution system, so several proposals are being developed in order to define and execute workflows based on BioMOBY services (as described in the next sub-section, Workflow management systems).

Semantic MOBY [37] is an extension created to solve the problems discovered in MOBY. This proposal defines four roles for software agents: Service Providers, Ontology Providers, Discovery Servers and Service Consumers. The architecture is based on an ontology that describes the relationships between these elements, and allows users to annotate services with different ontologies. The main idea in this proposal is to build RDF graphs with the service descriptions and to locate services using graph patterns. Thus, the main functionality it proposes is to match RDF graphs with user queries represented as graph patterns.

The National Institute for Bioinformatics (INB) in Spain has addressed the development of a Web client for locating and executing BioMOBY services [38]. The description of biological input/output objects is coordinated and standardized by means of a data type taxonomy in such a way that services can communicate with each other, wiring natural bioinformatics workflows. Automatic interfaces and help system builders have been incorporated into the architecture to make it more cohesive and to facilitate user communication. Beyond traditional bioinformatics platforms, data persistence systems, user management and scheduling abilities have produced a new generation of bioinformatics platforms.

#### Workflow management systems

The Taverna project [39] has developed a tool for the composition and execution of bioinformatics workflows. This tool includes a graphical interface for the creation and execution of workflows, which are described using a language called the Simple conceptual unified flow language (Scufl), where each step within a workflow represents one atomic task. This platform has been adapted in order to access BioMOBY services.

Remora [40] was designed to create and launch BioMoby workflows. The interface was simplified using the standard scheme of the traffic lights colour code (red, green and amber). It can only use its own workflow created by its own web interface. BIOWep [41] allows users to execute predefined workflows. It supports workflow annotation by using a simple ontology for bioinformatics processors (domain, task, i/o, etc.) and implements the search and selection of workflows on the basis of their annotation. It also supports retrieval of workflows on the basis of users' profiling. Biowep provides a semantic workflow repository. The user can search this repository using a graphical interface defining complex queries to find the desired workflow. Nowadays, this is the only portal/WMS that uses semantic annotation in the workflow systems.

GPIPE [42] offers a set of syntactic and algebraic operators which are able to represent analytical workflows in bioinformatics. Iteration, recursion, the use of conditional statements, and management of suspend/resume tasks have traditionally been implemented on an ad hoc and hard-coded basis. GPIPE is a prototype graphic pipeline generator for PISE that allows the definition of a pipeline, parameterization of its component methods, and storage of metadata in XML formats. GPIPE has been implemented and tested on the EMBOSS package. In order to employ other algorithms, it is necessary to describe the command-line user interface by means of XML. Thus, this proposal is only applicable to command-line applications, which should be in the local machine in which the workflow is going to be built and executed. It is not possible to use external applications published as web pages or web services.

BioWBI and WEE [43] have been designed to assist researchers in defining their data sources, drawing graphically and executing analysis workflows. These tools constitute the basic components of a much more general bioinformatics e-workplace, available via a web-browser that provides a collaborative space which is able to support both their analysis activities concerning the data management and the design and execution of their analysis processes. This proposal includes an XML description of algorithms, so their parameters are represented as data types. Thus, the only knowledge that a user needs to know in order to connect two applications is the parameter types, and it is not possible to check the consistency of the built workflow. This information would not be enough to build useful workflows, because parameters of the same type (strings for example) may have different semantics.

## Results

### Generic infrastructure

**Figure 1:**
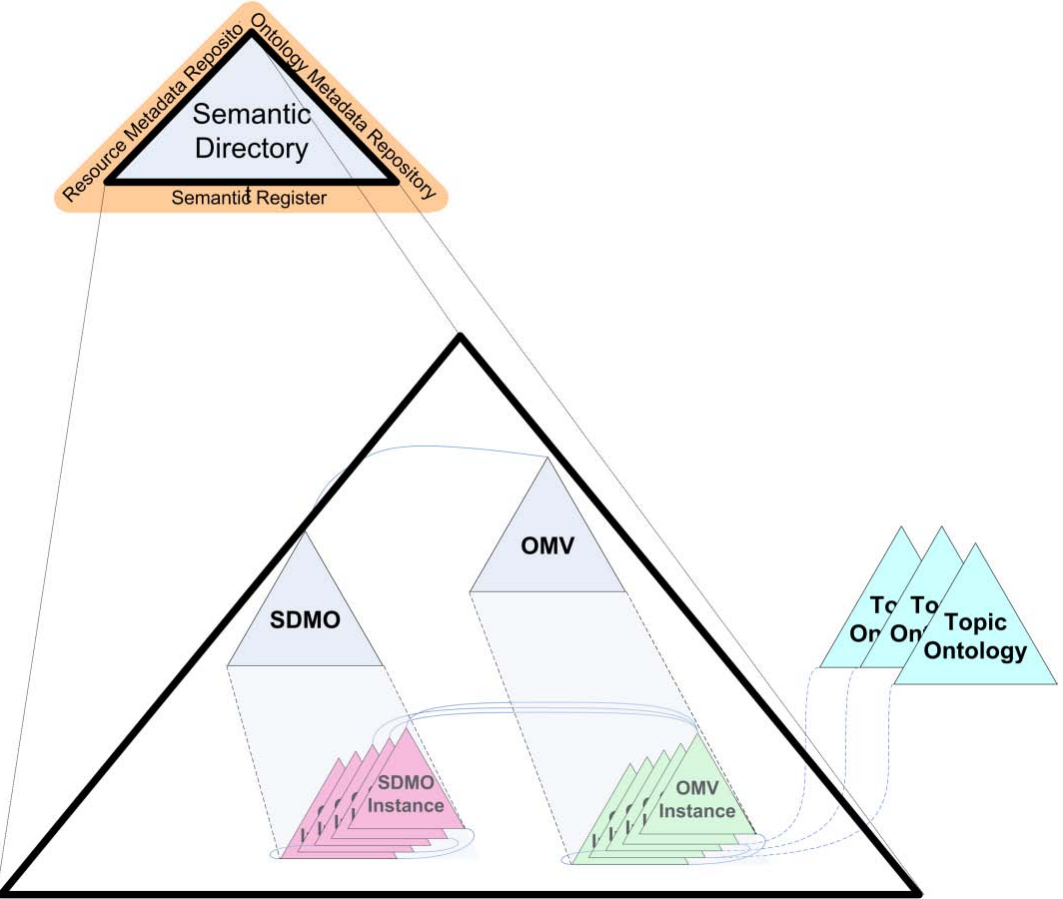
**Semantic Directory internal elements.** Two metadata ontologies provide the required elements to keep the metadata about registered ontologies (OMV: Ontology Metadata Vocabulary) and data sources (SDMO: Semantic Directory Metadata Ontology).

This section presents the generic infrastructure used as the core for the resolution of data integration problems in Systems Biology. This infrastructure is based on a resource directory, called Semantic Directory (Figure 1). We define the Semantic Directory as “a server to register semantics about available web resources, one or more registered ontologies, mappings between resources and these ontologies, and it provides services to browse all the registered semantics.”

The internal elements of the Semantic Directory are described by means of metadata. In order to deal with this metadata, the Semantic Directory is composed of two inter-related ontologies (OMV [44] and SDMO), which describe the internal semantics of the Semantic Directory (see Figure 1). This metadata can be managed by tools that can range from a simple OWL parser to a complex ontology reasoner. Nowadays, most of the ontologies are published without additional information such as who the owner is. This problem makes it difficult to identify, or to use and reuse published ontologies. For this reason we use OMV to register additional information about ontologies to help users locate and use them.

SDMO is the ontology in charge of registering information about resources and relationships between these resources and ontologies registered in the Semantic Directory. SDMO and OMV are related by a class included in SDMO, which provides a way of relating resources (SDMO instances) with registered ontologies (OMV instances). The current version of SDMO is composed of five classes:

• OMV: this class is used to link resources with registered ontologies (as instances of the OMV ontology).

• Resource: this class is used to store information (query capabilities, schema, query interface, name and URI) about resources.

• Mapping: this class is used to set the relationships between resources and ontologies. Each mapping is related with a similarity instance that establishes the similarity between ontology concepts and resource elements.

• Similarity: the similarity class contains three properties (concept1, concept2 and similarity Value) to establish the similarity between an ontology concept and a resource element. The similarity value is a real value between 0 and 1, indicating the probability of two concepts being the same. When adding manual mapping, the similarity value is 1, but if we use an automatic matching tool, this value may be less than 1. This similarity is used in the mediator to filter those mappings that are not taken into account in the query rewriting.

• User: this class is added in order to deal with users in the applications.

**Figure 2:**
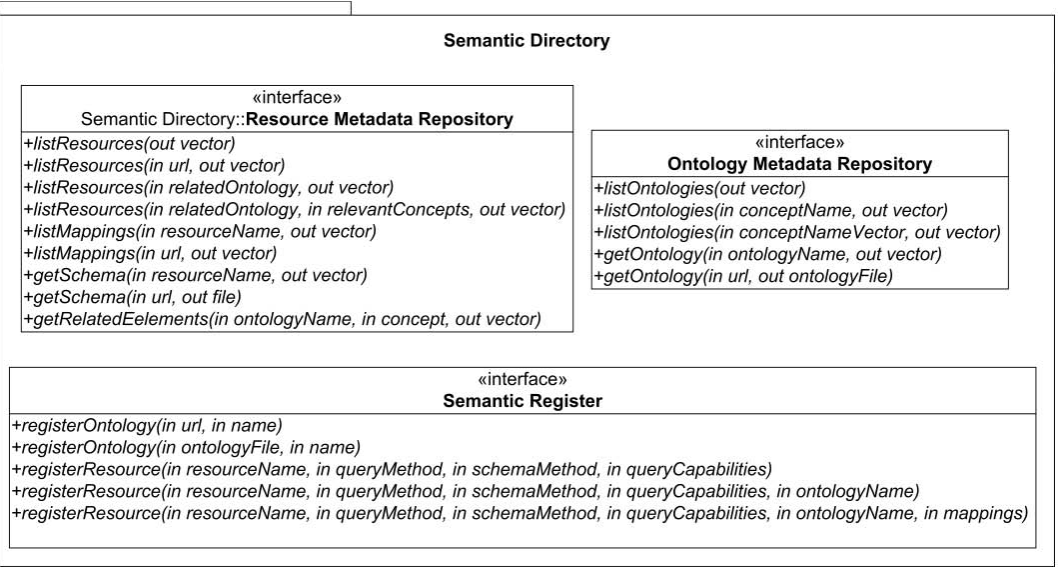
**Semantic Directories API**. Semantic Register is the interface in charge of providing methods for registering resources and ontologies in the semantic directory. Resource Metadata Repository provides methods for obtaining information about registered resources. Ontology Metadata Repository has several methods for obtaining information about registered ontologies.

The Semantic Directory provides three interfaces representing the main tasks it can perform: (1) Resource Metadata Repository; (2) Ontology Metadata Repository; and (3) Semantic Register. These components have been described by means of an API. This API has been represented as three Java interfaces (see Figure 2). This first implementation uses Jena to register and search for information. Registry methods are available to resource owners, and there are three possibilities:

º To make explicit the relationships with one of the ontologies registered in the Semantic Directory. These mappings will contain two parts: an expression in terms of the resource structure and an expression in terms of the ontology. The syntax of the mappings depends on the kind of resources registered and the applications developed over the semantic directory. Thus, a different kind of application needs a different extension of the semantic directory with a different mapping syntax. The next section presents an Ontology-Based Mediator which uses a Semantic Directory, so we will describe the syntax of the mappings for this case there.

º To take advantage of a tool for finding mappings between resource structures and one of the ontologies registered in the Semantic Directory. The first implementation contains a tool (Matching Tool, MaF) which can find mappings between XML documents and OWL ontologies, and between pairs of OWL ontologies. However, the Semantic Directory interface can be implemented using different tools.

º To take advantage of a tool for finding mappings between resource structures and all the ontologies registered in the Semantic Directory. The response time in this case depends on the number of ontologies registered and the tool used to compare structures.

Our goal is to provide applications which will make the semantics of the resources explicit through its commitment with an ontology registered in the Semantic Directory. The applications that can be developed using the Semantic Directory components depend on the extension of the infrastructure using new components (built on top of the Semantic Directory). Thus, semantic aware applications use the Semantic Directory to find the semantics of the resources registered in order to access the relevant information. These resources have to be registered in the Semantic Directory, but this will not involve making changes in them.

### Ontology-Based Mediator: an application example

As we propose the use of an ontology which is supposed to formalize a shared and consensus knowledge, the ontology used to integrate the data will be stable. For this reason, we have chosen a GAV approach. In GAV, each element in the data source schemas should be related with the terms of the integration schema.

**Figure 3:**
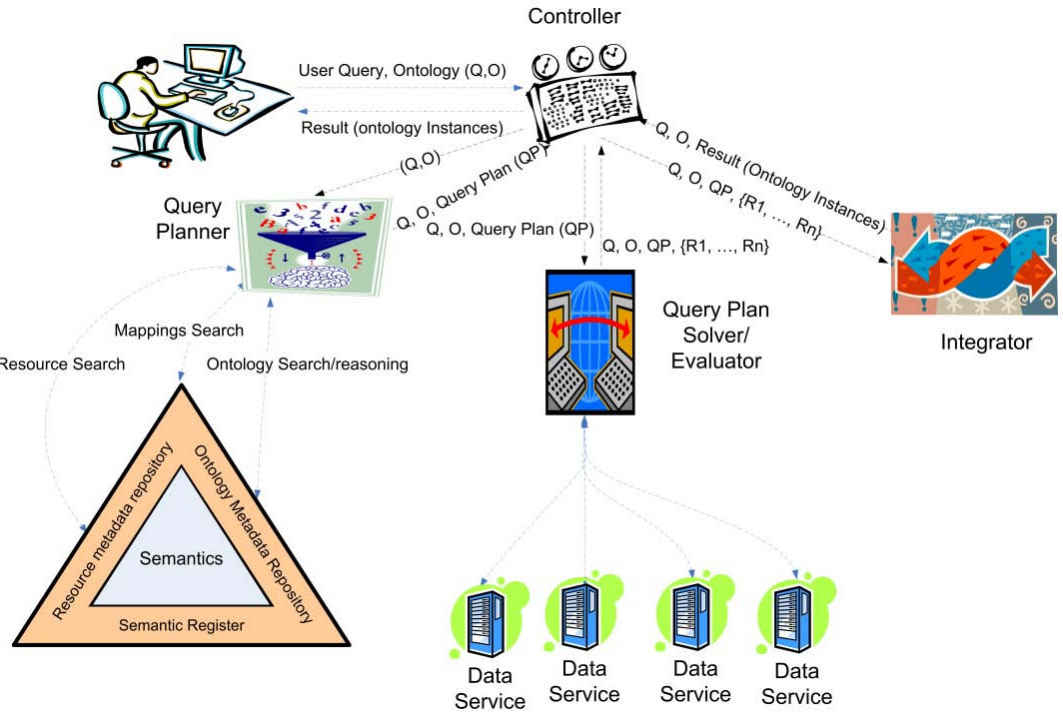
**Ontology-Base Mediator Architecture.** The mediator is composed of: a Controller (which controls the data flow in the system; a Query Planner in charge of finding a way of solving user queries using the Semantic Directory; a Query Plan Solver that executes the query plan making the corresponding calls to the data services; and an Integrator which integrates all the data retrieved from the data services to provide the user with the results of his/her query.

In order to benefit from the semantics, we have decided to develop an ontology-based mediator, which will take advantage of the generic infrastructure (described in the previous section) for dealing with semantics. Thus, we focus on a Mediation architecture that uses resources registered in a semantic directory. Registering of resources in the Semantic Directory is a key step towards the development of the integration solution, and this task is helped by ontologies. The architecture of the proposed Ontology-Based Mediator (Figure 3) is composed of four main components:

• *Controller*: the main task of this component is to interact with the user interface, providing solutions described in terms of one of the ontologies registered in the semantic directory.

• *Query Planner*: the task of this component is to find a query plan (*QP*) for the user query. The current planner has been implemented including the most basic reasoning mechanisms to take advantage of described semantics (subsumption and classification). Thus, if a query includes a concept this query will be expanded to include the semantic descendants. The mappings are also important in this process and they are used to find if the query pattern matches one or more patterns in the mappings. A bucket algorithm has been applied in this component, but we are working on more complex algorithms that could benefit more from reasoning mechanisms. Thus, more complex reasoning mechanisms can be included in this component, but considerable work still remains to be done to establish the consequences of its application.

• *Query Solver*: this component analyzes the query plan (*QP*), and performs the corresponding call to the data services involved in the sub-queries (*SQ_1_*, …,*SQ_n_*) of the query plan (*R_1_*, …,*R_n_*). This component will obtain a set of XML documents from different data services.

• *Integrator*: Results from data services (*R_1_*, …, *R_n_*) are composed by this component, in this way, obtaining the results of the user query. The current implementation of this component uses the mappings to translate the XML document to ontology instances, and then a conjunctive query evaluator is applied to the set of instances found. Future versions can include a reasoner, but this will imply taking care of the formal consequences of retrieving certain instances.

In our proposal (Figure 3), the sources are made available by publishing them as Web Services (named Data Services). Our primary goal here is to integrate databases accessible via internet pages. In this context, wrappers are an important part of the internal elements of data services. A wrapper is an interface to a data source that translates data into the common data model used by the mediator. In our case, we have chosen XML as the common data model. The development of Data Services that require the development of a wrapper has been studied in previous work [45]. However, biological data sources are usually public and downloadable. In these cases we have designed some patterns to retrieve a data source stored as a flat file to store it in an XML database. In summary, data services, independently of the development process, are distributed software applications that receive queries in XQuery and return XML documents.

In the context of mediator development, the process of registering resources in a semantic directory implies finding a set of mappings between one or several ontologies and the data service schema (usually expressed as an XMLSchema document). These mappings will be the key elements to integrate all the data sources, and these mapping will be the way in which the resource semantics are made explicit. The mappings used are defined as a pair (P,Q). P is a set of path expressions on the resource schema, and Q a query expression in terms of the ontology. In a first approach we have chosen XPath as the language to express P, and conjunctive queries to Q. For example to set the mappings between the Swiss-Prot data service and the ontology we have established the following mappings (registered in the Semantic Directory simply calling the register Resource method):

√ /Result/polypeptides/polypeptides_name

→ Polypeptides(P)

√ /Result/polypeptides/organism_name

→ Organism(O)

√ /Result/polypeptides/amino_acid_sequence

→ Amino_acids_Sequence(A)

√ /Result/polypeptides/polypeptides_name AND /Result/polypeptides/id

→ Polypeptides(P) AND SWISSPROT_id(P,i)

√ /Result/polypeptides/polypeptides_name AND /Result/polypeptides/molecular_weight

→ Polypeptides(P) AND molecular_weight(P,m)

√ /Result/polypeptides/polypeptides_name AND /Result/polypeptides/PDB

→ Polypeptides(P) AND PDB_id(P,i)

√ /Result/polypeptides/polypeptides_name AND /Result/polypeptides/synonym_polypeptides_name

→ Polypeptides(P) AND name_of_entities(P,n)

√ /Result/polypeptides/organism_name AND /Result/polypeptides/organism_name

→ Organism(O) AND Organism_name(O,n)

√ /Result/polypeptides/organism_name AND /Result/polypeptides/Taxon

→ Organism(O) AND Taxon_id(O,i)

√ /Result/polypeptides/amino_acid_sequence AND /Result/polypeptides/ amino_acid_sequence

→ Amino_acids_Sequence(A) AND sequence(A,s)

√ /Result/polypeptides/amino_acid_sequence AND /Result/polypeptides/ length_sequence

→ Amino_acids_Sequence(A) AND length(A,l)

√ /Result/polypeptides/polypeptides_name AND /Result/polypeptides/ organism_name

→ belong_to(P,O)

√ /Result/polypeptides/polypeptides_name AND /Result/polypeptides/ amino_acid_sequence

→ amino_acids_sequence(P,A)

### The Amine System Project: integration of data on biological amine-related information

In this pilot project (*ASP Model Finder*[14]) we have developed and tested the Ontology-Based Mediator described in the previous section. The long term goal of this project is to register the most relevant public databases at different levels of study: metabolite properties and concentrations, macromolecular structures, assigned functions, docking among biomolecules and information on biochemical pathways. This proposal is flexible as has been shown in the previous section, but in order to validate its viability for solving specific and real issues, we have tackled the resolution of a well known bioinformatics problem by integrating a limited but increasingly growing number of databases. The initial problem to be solved is summarized as following, and the use case developed to solve it is named AMMO-Prot:

*Problem*: A common and useful strategy to determine the 3D structure of a protein, which cannot be obtained by its crystallization, is to apply comparative modelling techniques. These techniques start working with the primary sequence of the target protein to finally predict its 3D structure by comparing the target polypeptide to those of solved homologous proteins [46].

**Figure 4:**
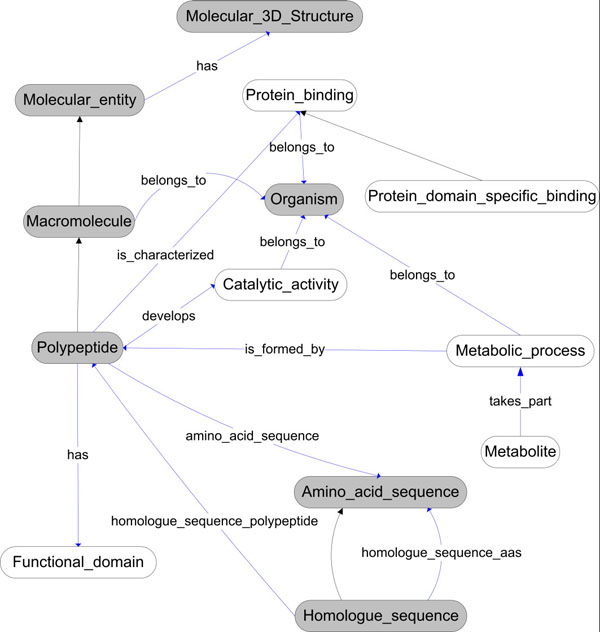
**AMMO Ontology**. This ontology represents the key concepts in the use case we present below and other on-going use cases. Nodes are concepts, unlabeled arrows are “is_a” relationships (concept hierarchy), and labelled arrows are the properties defined to relate concepts. The main concepts in the example presented in this paper are Organism, Polypeptides, Homologue_Sequence, Amino_acids_Sequence and Molecular_3D_Structure.

In order to solve this problem, protein structure data and some tools to compare them are required from databases. These databases have been used to validate our integration tool. First of all, we need to define the domain ontology in order to relate it to the resource semantics. There are several ontologies related to this domain, the most representative being GO () and the molecular biology ontology TAMBIS (). However, these ontologies are very large and describe very light weight semantics (only describing concept hierarchy). Thus, we have developed our own ontology (The AMine Metabolism Ontology, AMMO) which is based on the Gene Ontology concepts but with enriched relationships which improve its semantics (see Figure 4). The definition of the relationships between concepts will allow us to retrieve interrelated information (for example polypeptides and the organisms in which they can be found). In addition, future versions of the mediator will benefit from the improved semantics to infer new knowledge.

Being based on the GO ontology guarantees interoperability with other applications also based on this ontology. In Figure 4, we have shown only the relevant concepts for our domain helping users to understand the domain semantics. The AMMO is used as the pivot that will integrate the whole domain, which includes concepts/relationships necessary for the use case described above and other ongoing projects. This ontology has been described with OWL.

At present, the AMMO ontology has been used to register the semantics of different consolidated resources/tools in bioinformatics: SWISS-Prot [47], PDB [48], Modeller [49] and JMol (). They allow us to retrieve information about protein structures which is used at an initial analysis stage. This information will subsequently be the basis of future developments to extend the possibilities of our system, and to allow users to retrieve information on many other queries on metabolic, signalling and molecular interactions and relationships i.e., to further progress in automatic data integration resources on a given biochemical problem. In order to achieve this goal new databases (PubChem, Kegg, Brenda and Prosite) are being wrapped.

**Figure 5:**
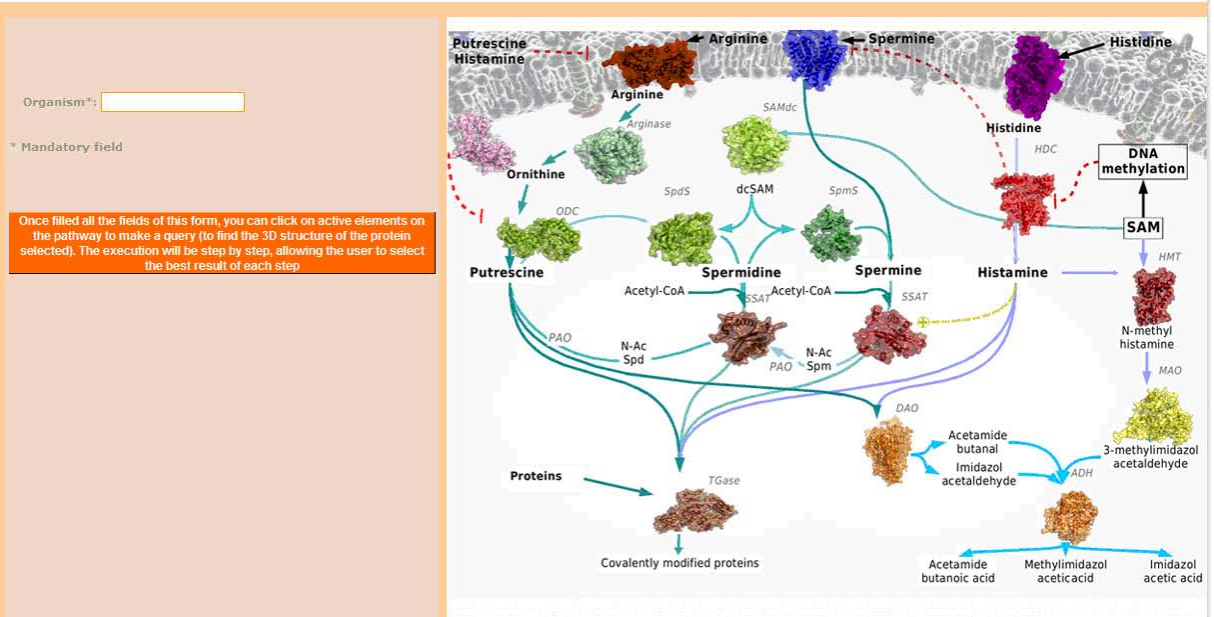
**AMMO-Prot interface.** The pathway is used as the entry point in the developed use case (). Each protein/enzyme is a link to a workflow that will capture the available structural information on the polypeptide in a given species.

In its present stage, the developed Web tool has as its goal to show the viability of the proposal and its application in the real case mentioned above (location/prediction of an amine-related protein structure), so we have divided the problem into a set of simple steps. Initially, the species must be selected by filling its (common or scientific) names in the “organism” field (Figure 5). On the other hand, the Web interface shows a pathway that is used as the entry point (Figure 5) to retrieve structural information on the target by clicking on the protein picture. The process for information searching and integration is illustrated by the two examples of human polypeptides explained below.

In the first example, we click on the enzyme “SSAT (Spermidine/Spermine N-acetyltransferase)”(synonymous of “diamine acetyltransferase”). Thus, the first query to be solved is:

*P* ← *Polypeptide(P) and name(P*, *“Spermidine/Spermine N-acetyltransferase”) and Organism(O) and name(O*, *“Human”) and belongs_to(P,O)*.

The query built inside the Web application is decomposed into two sub-queries to be sent to the Swiss-Prot and PDB databases, which are the databases related to the polypeptide concept. First the sub-query will return data as an instance of the Polypeptide concept. Thus, the query results comprise information available in the Swiss-Prot database on two isoforms for the human SSAT protein. Then, the graphical Web interface will allow the users to manually select from information (including the primary sequence) for one or another isoform. In this specific case, both structures have been previously determined by experimental methods, so that the application will make use of the second sub-query to retrieve information from the PDB database, which could be downloaded or displayed using the JMol tool (). When launching a new query about an unsolved structure (or one not annotated in PDB), as is the case for “TGase (Protein-glutamine gamma-glutamyltransferase),” an initial search for information about this enzyme would start in the SwissProt database as in the previous example. Results will show all the entries related with our query, which correspond with different tissue-specific TGase types. In this task, we are going to focus on TGase Z, which is widely expressed in humans. As there is no entry in PDB for this protein, the next step involves executing the Modeller tool [49] to predict a 3D structure of the polypeptide. Modeller is an automated homology modelling tool, which performs the necessary steps to carry out: searching for homologous proteins, target-template sequence alignments, model building on the bases of the template coordinates and basic geometry optimization. In our example (TGase Z) the user query for this process is:

*Q(HS): Polypeptide (P1) and Amino_acid_sequence(AA) and sequence (AA*, *“MDQVATLRLESV…”) and amino_acid_sequence (P1,AA) and Polypeptide (P2) and Homologue_Sequence (HS) and homologue_sequence_aas(HS,AA) and homologue_sequence_polypeptide(HS*,*P2)*.

Then, using the primary sequence retrieved from SwissProt, a search on PDB is performed to obtain a set of homologous proteins of our target, corresponding to structures that have been previously determined experimentally by using mainly X-ray diffraction or NMR techniques. We obtain a set of template candidates that are filtered by the sequence identity shared with the target sequence. In a homology modelling process the quality of the final model is highly dependent on the identity between target and template sequences, so those templates sharing an identity below 30% are going to be discarded by the application [46]. In this first version, the template showing the highest identity with the target protein will be selected for the next steps. Once the most suitable template is chosen, further steps of alignment and modelling are performed by the automated tool. Template and target sequences are aligned before the modelling process, where spatial coordinates of the template are extrapolated to build the target structure.

**Figure 6:**
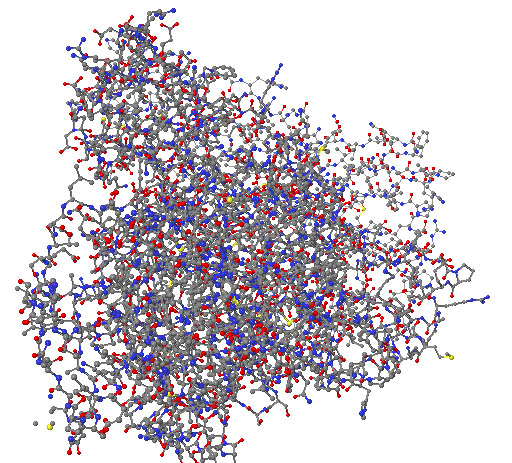
**3D model predicted by AMMO-Prot for human TGase Z.** Figure created with JMol [].

Finally, the results of this process are five different models of the same target protein, which can be selected in the Web interface. Subsequently, the model can be either downloaded or viewed using JMol, in order to analyze whether the solution is realistic or not (Figure 6). This visualization tool is included as part of the application, providing added-value features.

## Discussion and conclusions

This paper presents an Ontology-Based Mediator that uses an infrastructure for building applications in the Semantic Web. It is being validated in a specific biological context, that of amine-related biochemistry, and consequently it has been named the Amine System Project (). Of course, this Semantic Mediator can be adapted very easily to obtain structural information for many other biochemical problems as can be deduced form the AMMO ontology organization (Figure 4). The proposed ontology is an extension of the Gene Ontology, so the developed system can interoperate with other systems using this ontology (using shared vocabulary). If the AMMO ontology is modified to add new concepts, existing mappings will still be valid, ensuring system scalability. However, if a concept is changed, related mappings should be redefined. In addition, the developed proposal is a generic infrastructure and mediator, so the ontology used can be changed to be used in other domains (adding new mappings between resources and registered ontologies). In the development of the mediator and in previous works [34][37][45], we have discovered some limitations. The main one is the maintenance of data services, because the services developed use public databases that are not under our control. Thus, the long term success of this proposal and similar ones relies on the collaboration of data and tool owners. For this reason, the data services integrated into this proposal have been developed from stable data sources, providing their data as files, data services or databases (system that are not affected by aspect changes, as in web interfaces). In addition, the developed data services include the use of internal cache systems to prevent source unavailability.

The proposed solution is based on the registration of the resources' semantics by relating them with ontologies. However, the location of relevant ontologies in a specific domain is an open problem which is being dealt with by relating ontologies (ontology alignment) and organizing them. In this way, we are studying how to extend our proposal to include mechanisms for organizing ontologies in order to facilitate their location.

Protein structures contain fundamental information regarding their function, location and interactions, which is most of the information in their biological missions. Our use case (AMMO-Prot) returns the correct information for the protein structures included initially in the pilot project. The pilot could be easily adapted to any other metabolic pathway. In addition, queries can be defined in a user friendly way for biochemists, that is, by clicking on the protein symbols organized as a metabolic pathway scheme after definition of the required species.

Combining information integration with prediction techniques results in efficient information retrieval and expands the spectrum of applicability of structural bioinformatics techniques to non-experienced users. Therefore, the problem presented in this paper is important in this context, as automatic performance of the process will reduce the effort required to solve questions on protein structures. All of these characteristics will expand the spectrum of applicability of structural bioinformatics techniques to non-experienced users. Genomic Projects have exponentially increased the number of known polypeptide sequences. Thus, any effort to improve efficiency for the extraction of structural information at its highest level should help advance many on-going Systems Biology projects. Our project fulfils all of these aims and objectives.

## List of abbreviations used

ASP: Amine System Project

AMMO: the Amine Metabolism Ontology

KEGG: Kyoto Encyclopedia of Genes and Genomes

LAV: Local As View

GAV: Global As View

TSIMMIS: The StanfordIBM Manager of Multiple Information Sources

XML: eXtensible Markup Language

MOCHA: Middleware Based On a Code SHipping Architecture

TAMBIS: Transparent Access to Multiple Bioinformatics Information Sources

RDF: Resource Description Framework

INB: National Institute for Bioinformatics

BIOWeP: Workflow Enactment Portal for Bioinformatics

WMS: Workflow Management System

PISE: Pasteur Institute Software Environment

EMBOSS: European Molecular Biology Open Software Suite

BioWBI: Bioinformatic Workflow Builder Interface

WEE: Workflow Execution Engine

OMV: Ontology Metadata Vocabulary

SDMO: Semantic Directory Metadata Ontology

OWL: Web Ontology Language

URI: Uniform Resource Identifier

API: Application Programming Interface

MaF: Matching Framework

XQuery: XML Query Language

XPath: XML Path Language

GO: Gene Ontology

PDB: Protein Data Bank

## Competing interests

The authors declare that they have no competing interests.

## Authors' contributions

IND designed the infrastructure of Semantic Directories, carried out the implementation of the system and drafted the manuscript. RM designed the biological problem to be solved. RM and APA carried out the study of the databases and analyzed result provided by AMMO-Prot. AMG developed the study of mechanisms to predict structures. JLU and FSJ participated in the design of the study, coordinated the test phases and performed analysis of the results. FSJ and helped to draft the manuscript. JFAM conceived the infrastructure, participated in its design and coordination and helped to draft the manuscript. All authors read and approved the final manuscript.

## References

[B1] Kitano H (2002). Systems biology: a brief overview. Science.

[B2] Viguera E, Trelles O, Urdiales JL, Matés JM, Sánchez-Jiménez F (1994). Mammalian L-amino acid decarboxylases producing 1,4-diamines: analogies among differences. Trends Biochem Sci.

[B3] Rodríguez-Caso C, Rodríguez-Agudo D, Moya-García A, Fajardo I, Medina MA, Subramaniam V, Sánchez-Jiménez F (2003). Local changes in the catalytic site of mammalian histidine decarboxylase can affect its global conformation and stability. Eur J Biochem.

[B4] Rodríguez-Caso C, Montañez R, Cascante M, Sánchez-Jiménez F, Medina MA (2006). Mathematical modeling of polyamine metabolism in mammals. J Biol Chem.

[B5] Medina MA, Urdiales JL, Matés JM, Núñez de Castro I, Sánchez-Jiménez F (1991). Diamines interfere with the transport of L-ornithine in Ehrlich-cell plasma-membrane vesicles. Biochem J.

[B6] Medina MA, Quesada AR, Núñez de Castro I, Sánchez-Jiménez F (1999). Histamine, polyamines and cancer. Biochem Pharmacol.

[B7] Medina MA, Urdiales JL, Rodríguez-Caso C, Ramírez FJ, Sánchez-Jiménez F (2003). Biogenic amines and polyamines: similar biochemistry for different physiological missions and biomedical applications. Crit Rev Biochem Mol Biol.

[B8] Moya-García AA, Medina MA, Sánchez-Jiménez F (2005). Mammalian histidine decarboxylase: from structure to function. BioEssays.

[B9] Urdiales JL, Matés JM, Núñez de Castro I, Sánchez-Jiménez F (1992). Chlorpheniramine inhibits the ornithine decarboxylase induction of Ehrlich carcinoma growing in vivo. FEBS Lett.

[B10] Dopazo J (2006). Bioinformatics and cancer: an essential alliance. Clin Transl Oncol.

[B11] Overall CM, Dean RA (2006). Degradomics: systems biology of the protease web. Pleiotropic roles of MMPs in cancer Cancer Metastasis Rev.

[B12] Liu ET, Kuznetsov VA, Miller LD (2006). In the pursuit of complexity: systems medicine in cancer biology. Cancer Cell.

[B13] Carlberg C, Dunlop TW (2006). An integrated biological approach to nuclear receptor signaling in physiological control and disease. Crit Rev Eukaryot Gene Expr.

[B14] AMMO-Prot. http://asp.uma.es/WebMediator.

[B15] Brogi A, Corfini S, Aldana-Montes JF, Navas-Delgado I, Georgakopoulos D, Ritter N, Benatallah B, Zirpins C, Feuerlicht G, Schoenherr M, Motahari-Nezhad HR (2006). Automated Discovery of Compositions of Services Described with Separate Ontologies. Proceedings of the 4th International Conference on Service Oriented Computing: 4-7 December 2006; Chicago.

[B16] Navas-Delgado I, Sanz I, Aldana-Montes JF, Berlanga Llavori R, Andersen KV, Debenham J, Wagner R (2005). Automatic Generation of Semantic Fields for Resource Discovery in the Semantic Web. Proceedings of the 16th International Conference on Database and Expert Systems Applications: 22-26 August 2005; Copenhagen.

[B17] Halevy AY (2001). Answering queries using views: A survey. VLDB Journal: Very Large Data Bases.

[B18] Grahne G (2002). Information integration and incomplete information. IEEE Data Eng Bull.

[B19] Ullman JD (2000). Information integration using logical views. Theoretical Computer Science.

[B20] Levy AY, Minker J (1999). Logic-based techniques in data integration. Proceedings of the Workshop on Logic-Based Artificial Intelligence: 14-16 June 1999; Washington, DC.

[B21] Lenzerini M, Popa L (2002). Data integration: A theoretical perspective. Proceedings of the 21st ACM SIGACT-SIGMOD-SIGART Symposium on Principles of Database Systems: 3-6 June 2002; Madison.

[B22] Bertossi L, Chomicki J, Chomicki J, Meyden R, Saake G (2003). Query answering in inconsistent databases. Logics for Emerging Applications of Databases.

[B23] Chawathe SS, Garcia-Molina H, Widom J (1994). Flexible constraint management for autonomous distributed databases. Data Engineering Bulletin.

[B24] Levy A (1998). The information manifold approach to data integration. IEEE Intelligent Systems.

[B25] Bornhovd C (1998). Mix - a representation model for the integration of web-based data. Tech Rep.

[B26] Rodriguez-Martinez M, Roussopoulos N, Chen W, Naughton JF, Bernstein PA (2000). Mocha: a self-extensible database middleware system for distributed data sources. Proceedings of the ACM SIGMOD International Conference on Management of Data: 16-18 May 2000; Dallas.

[B27] Mena E, Kashyap V, Illarramendi A, Sheth A (2000). Imprecise answers on highly open and distributed environments: An approach based on information loss for multiontology based query processing. International Journal of Cooperative Information Systems (IJCIS).

[B28] Ascher L, Gupta B, Martone A, Critchlow T, Lacroix Z (2003). A model-based mediator system for scientific data management. Bioinformatics: Managing Scientific Data.

[B29] Stevens R, Baker P, Bechhofer S, Ng G, Jacoby A, Paton NW, Goble CA, Brass A (2000). TAMBIS: Transparent access to multiple bioinformatics information sources. Bioinformatics.

[B30] Lange M, Freier A, Scholz U, Stephanik A, Wingender E (2001). A computational support for access to integrated molecular biology data. Proceeding of the German Conference on Bioinformatics: 7-10 October 2001; Braunschweig.

[B31] Gupta A, Ludäscher B, Martone ME, Günther O, Lenz HJ (2000). Knowledge-based integration of neuroscience data sources. Proceedings of the 12th International Conference on Scientific and Statistical Database Management (SSDBM): 26-28 July 2000; Berlin.

[B32] Liu L, Buttler D, Critchlow T, Han W, Paques H, Pu C, Rocco D, IEEE Computer Society Press (2003). BioZoom: Exploiting source-capability information for integrated access to multiple bioinformatics data sources. Proceedings of the 3rd IEEE Symposium on BioInformatics and BioEngineering: 10-12 March 2003; Washington, DC.

[B33] Davidson S, Overton C, Tannen V, Wong L (1997). BioKleisli: A digital library for biomedical researchers. International Journal of Digital Libraries.

[B34] IBM Corp. DiscoveryLink.

[B35] Aldana JF, Roldán-Castro M, Navas-Delgado I, Roldán-García MM, Hidalgo-Conde M, Trelles O (2006). Bio-Broker: a tool for integration of biological data sources and data analysis tools. Software: Practice and Experience.

[B36] Wilkinson MD, Gessler D, Farmer A, Stein L (2003). The Bio-MOBY Project Explores Open-Source, Simple, Extensible Protocols for Enabling Biological Database Inter-operability. Proceedings of the Virtual Conference Genomic and Bioinformatics: 16-19 September 2003.

[B37] Schiltz G, Gessler D, Stein L, W3C (2004). Semantic MOBY. Proceedings of the W3C Workshop on Semantic Web for Life Sciences: 27-28 October 2004.

[B38] Navas-Delgado I, Rojano-Muñoz MM, Ramírez S, Pérez AJ, Andrés León E, Aldana-Montes JF, Trelles O (2006). Intelligent client for integrating bioinformatics services. Bioinformatics.

[B39] Oinn T, Addis M, Ferris J, Marvin D, Senger M, Greenwood M, Carver T, Glover K, Pocock MR, Wipat A, Li P (2004). Taverna: A tool for the composition and enactment of bioinformatics workflows. Bioinformatics.

[B40] Carrere S, Gouzy J (2006). REMORA: a pilot in the ocean of BioMoby web-services. Bioinformatics.

[B41] Romano P, Bartocci E, Bertolini G, De Paoli F, Marra D, Mauri G, Merelli E, Milanesi L (2007). Biowep: a workflow enactment portal for bioinformatics applications. BMC Bioinformatics.

[B42] Garcia Castro A, Thoraval S, Garcia LJ, Ragan MA (2005). Workflows in bioinformatics: meta-analysis and prototype implementation of a workflow generator. BMC Bioinformatics.

[B43] Leo P, Marinelli C, Pappadà G, Scioscia G, Zanchetta L (2004). BioWBI: an Integrated Tool for building and executing Bioinformatic Analysis Workflows. Bioinformatics Italian Society Meeting: 26-27 March 2004; Padova.

[B44] Hartmann J, Sure Y, Haase P, Palma R, Suarez-Figueroa M, Gil Y, Motta E, Benjamins VR, Musen MA (2005). Omv - ontology metadata vocabulary. Proceedings of the 4th International Semantic Web Conference: 6-10 November 2005; Galway.

[B45] Navas Delgado I, Roldán-García MM, Dianes Mazorra D, Aldana-Montes JF, Castro J, Teniente E (2005). Developing Data Services. Proceegings of the 17th Conference on Advanced Information Systems Engineering Data Integration and the Semantic Web: 13-17 June 2005; Porto.

[B46] Baker D, Sali A (2001). Protein structure prediction and structural genomics. Science Date.

[B47] Boeckmann B, Bairoch A, Apweiler R, Blatter MC, Estreicher A, Gasteiger E, Martin MJ, Michoud K, O'Donovan C, Phan I, Pilbout S, Schneider M (2003). The swiss-prot protein knowledgebase and its supplement trembl in 2003. Nucleic Acids Res.

[B48] Kellenberger E, Muller P, Schalon C, Bret G, Foata N, Rognan D (2006). sc-pdb: an annotated database of druggable binding sites from the protein data bank. J Chem Inf Model.

[B49] Marti-Renom MA, Stuart A, Fiser A, Sánchez R, Melo F, Sali A (2000). Comparative protein structure modeling of genes and genomes. Annu Rev Biophys Biomol Struct.

